# Unveiling the Promoting Mechanism of H_2_ Activation on CuFeO_x_ Catalyst for Low-Temperature CO Oxidation

**DOI:** 10.3390/molecules29143347

**Published:** 2024-07-17

**Authors:** Zhenghua Shen, Xiangdong Xing, Yuan She, Hao Meng, Wenkang Niu, Shan Ren

**Affiliations:** 1 School of Metallurgical Engineering, Xi’an University of Architecture and Technology, Xi’an 710064, China; shenzhenghhua@xauat.edu.cn (Z.S.); xaxxd@xauat.edu.cn (X.X.); mhao0325@163.com (H.M.); n18165212819@163.com (W.N.); 2 Metallurgical Engineering Technology Research Center of Shaanxi Province, Xi’an 710055, China; 3 College of Materials Science and Engineering, Chongqing University, Chongqing 400044, China

**Keywords:** CO oxidation, CuFeO_x_ catalyst, H_2_ activation, Cu^+^ species

## Abstract

The effect of H_2_ activation on the performance of CuFeO_x_ catalyst for low-temperature CO oxidation was investigated. The characterizations of XRD, XPS, H_2_-TPR, O_2_-TPD, and in situ DRIFTS were employed to establish the relationship between physicochemical property and catalytic activity. The results showed that the CuFeO_x_ catalyst activated with H_2_ at 100 °C displayed higher performance, which achieved 99.6% CO conversion at 175 °C. In addition, the H_2_ activation promoted the generation of Fe^2+^ species, and more oxygen vacancy could be formation with higher concentration of O_α_ species, which improved the migration rate of oxygen species in the reaction process. Furthermore, the reducibility of the catalyst was enhanced significantly, which increased the low-temperature activity. Moreover, the in situ DRIFTS experiments revealed that the reaction pathway of CO oxidation followed MvK mechanism at low temperature (<175 °C), and both MvK and L-H mechanism was involved at high temperature. The Cu^+^-CO and carbonate species were the main reactive intermediates, and the H_2_ activation increased the concentration of Cu^+^ species and accelerated the decomposition carbonate species, thus improving the catalytic performance effectively.

## 1. Introduction 

The incomplete combustion of fossil fuels results in industrial flue gases containing certain concentrations of CO. For example, the low-temperature flue gas (80–180 °C) generated during the iron ore sintering process contains 0.5–2% CO [1,2]. The ability of CO to bind to hemoglobin is stronger than oxygen. The direct release of CO is detrimental to human health and causes various environmental issues, including the degradation of the ozone layer [3,4,5]. Catalytic oxidation could efficiently oxidize CO into nontoxic CO_2_ with minimal energy consumption, which is considered to be the most promising CO elimination technology at present [6,7]. Au, Pt, and other precious metal catalysts exhibit high catalytic activity [8]. Nevertheless, the elevated expense associated with precious metal catalysts poses a constraint on the widespread deployment of CO oxidation in large-scale industrial flue gas treatment. It is necessary to develop a catalytic material with low price and excellent performance for CO oxidation.

The Cu-based catalysts display a great development prospect in CO oxidation due to the unique CO adsorption and activation property [9,10]. However, the pure CuO catalyst has poor activity at low temperature. The catalyst of CuO combined with CeO_2_, Fe_2_O_3,_ Co_3_O_4_, and MnO_2_ shows high catalytic performance at low temperatures [11,12,13,14,15]. Notably, the CuFeO_x_ catalysts display exceptional performance in CO oxidation, attributed to the remarkable oxygen storage capacity and Fe^2+^/Fe^3+^ redox cycle inherent in Fe_2_O_3_ [16,17,18]. This suggests a potential for CuFeO_x_ catalysts to supplant precious metal catalysts in CO removal from industrial flue gas. According to our previous research [19,20], the structure and oxidation state of the active components have significant effects on the catalytic performance. Previous studies have shown that the pretreatment and activation could adjust the structure and the content of oxidation states of active components of catalysts [21,22,23], which could effectively improve the catalytic performance. Wang et al. [21] reported that the H_2_ pretreated Pd/Fe_2_O_3_ catalyst exhibited higher catalytic activity due to the higher concentration of Fe^2+^ species and stronger reducibility. Wang et al. [22] explored the impact of H_2_ activation on the catalytic activity of the CuCeO_x_ catalyst. The H_2_ pretreatment increased the amount of highly dispersed CuO_x_ and oxygen vacancy, which enhanced the activity of CO oxidation. This showed us that the performance of the CuFeO_x_ catalyst may be improved by H_2_ pretreatment. However, the effect of H_2_ pretreatment on the performance of the CuFeO_x_ catalyst has seldom been explored in detail.

In this work, the CuFeO_x_ catalyst was prepared and activated with H_2_ at different temperatures to investigate the influence of pretreatment on the performance. In addition, the XRD, BET, XPS, H_2_-TPR, and O_2_-TPD were characterized to establish the relationship between structure property and catalytic performance. Furthermore, the reaction mechanism was revealed by in situ DRIFTS studies. This work may provide a novel strategy for the improvement of performance of the CuFeO_x_ catalyst.

## 2. Results and Discussion

### 2.1. Catalytic Performance

**Figure 1:**
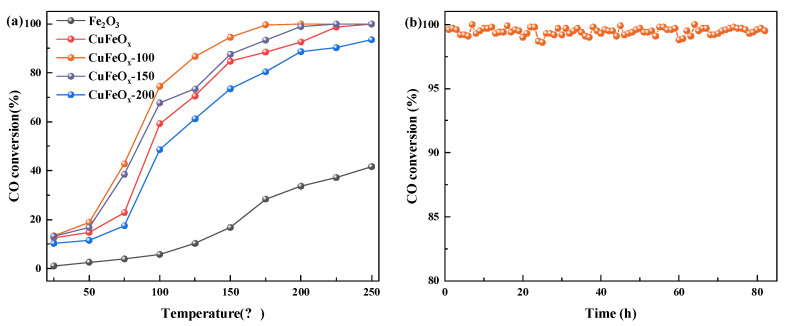
(**a**) CO conversion of all catalysts. (**b**) The stability of CuFeO_x_-100 catalyst.

The activity of catalysts was measured and is displayed in Figure 1a. It was observed that the CO conversion increased with increasing temperature for all catalysts. As for pure Fe_2_O_3_, the CO conversion was only 41.7% at 250 °C. As expected, the activity was improved after the addition of Cu species, and 100% CO conversion could be achieved at 250 °C for the CuFeO_x_ catalyst. In addition, the activity of CuFeO_x_ and H_2_-activated catalysts was insignificantly different at low temperature, while the activities of CuFeO_x_-100 and CuFeO_x_-150 catalysts were higher than CuFeO_x_ catalysts after 75 °C. The CuFeO_x_-100 catalyst displayed the highest activity with 99.6% CO conversion at 175 °C, which indicated that the appropriate activation temperature could effectively increase the catalytic performance. However, it could be noted that the CO conversion of the CuFeO_x_-200 catalyst was obviously lower than the CuFeO_x_ catalyst, which may be due to the over-reduction decreasing the active species. The service life is another important factor in evaluating catalyst applications. The stability of the CuFeO_x_-100 catalyst was measured and is shown in Figure 1b. The reaction continued for 82 h at 175 °C, keeping the composition and airspeed of the gas mixture unchanged. It can be seen that the CO conversion almost did not decrease after 82 h, and displayed excellent stability.

### 2.2. Structural and Textural Properties

**Figure 2:**
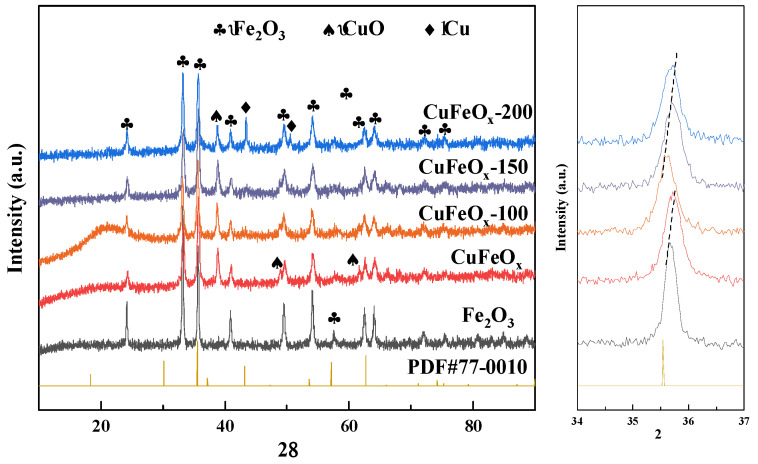
XRD patterns of catalysts.

The crystal structure of the catalysts was investigated with XRD and is depicted in Figure 2. The pure Fe_2_O_3_ displayed the diffraction peaks at 24.2°, 33.2°, 35.6°, 40.9°, 49.5°, 54.1°, 57.6°, 62.4°, 64.0°, 71.9°, and 75.5°, which corresponded to the crystallized α-Fe_2_O_3_ for (012), (104), (110), (113), (024), (116), (122), (214), (300), (101), and (220), respectively. The diffraction peaks of pure Fe_2_O_3_ presented with high crystallinity. As for the CuFeO_x_ catalyst, new peaks attributed to CuO at 38.8°, 48.6°, and 61.6° appeared. In addition, the peak intensity of Fe_2_O_3_ decreased significantly compared with pure Fe_2_O_3_, indicating the formation of a strong interaction between Fe and Cu species, as a result of the smaller particle size and reduced crystallinity. For CuFeO_x_-100 and CuFeO_x_-150 catalysts, the peaks at 48.6° and 61.6° weakened and even disappeared, possibly attributable to the reduction of CuO to Cu_2_O and/or metallic Cu. The peaks assigned to metallic copper at 43.4° and 50.5° could be found on the CuFeO_x_-200 catalyst, which confirmed that part of the CuO species was reduced to metallic Cu species due to the H_2_ activation. Interestingly, the peaks of Fe_2_O_3_ at 35.7° on the H_2_-activated catalysts shifted to a lower 2θ degree compared to the CuFeO_x_ catalyst. This demonstrated that the H_2_ activation promoted the formation of Fe^2+^ species and resulted in the lattice expansion, which suggested that the Fe_2_O_3_ also participated in the reduction process. In addition, no obvious diffraction peaks related to the CuFeOx phase were found.

The N_2_ adsorption–desorption isotherms are displayed in Figure S1, and the physical property of the catalysts is shown in Table 1. As shown in Figure S1, all catalysts presented type IV isotherms and H3 type hysteresis loops, which suggests the existence of mesopores. The mesopores were conducive to the dispersion of active particles and the diffusion of reactive gases. The specific surface area, average pore diameter, and total pore volume of pure Fe_2_O_3_ was 9.85 m^2^/g, 14.96 nm, and 0.037 cm^3^/g, respectively. After the addition of the Cu species, the specific surface area and total pore volume of the CuFeO_x_ catalyst increased slightly, while the average pore diameter was reduced. This suggests that the strong interaction among Fe and Cu species hindered the growth of particles, thereby enhancing the dispersion of active components, and more active sites could be provided for CO oxidation. In addition, the difference in specific surface area, average pore diameter, and total pore volume between CuFeO_x_ and CuFeO_x_-100 catalysts could be negligible, which demonstrates that the influence of H_2_ activation on the surface structure of the catalyst was less. The CuFeO_x_ and CuFeO_x_-100 catalysts had similar surface structure, while the activity of the CuFeO_x_-100 catalyst was obviously higher than the CuFeO_x_ catalyst. This suggests that the surface physical properties were not the determining factor for the increased activity of the CuFeO_x_-100 catalyst. The morphological features of the catalysts were investigated with SEM, as displayed in Figure S2. The catalysts existed in irregular flakes and particles of various sizes. The surface morphology of the catalysts had no obvious change after H_2_ activation.

### 2.3. Surface Chemical States

**Figure 3:**
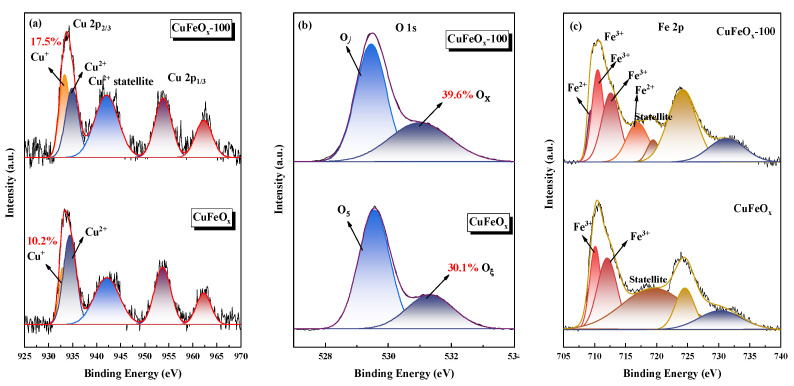
XPS spectra of catalysts: (**a**) Cu 2p; (**b**) O 1s; and (**c**) Fe 2p.

The effect of H_2_ activation on surface chemical composition on the catalysts was determined with XPS, as depicted in Figure 3. In addition, the atomic percentages of elements were calculated from the areas of the fitted peaks and were presented in Table 2. The Cu 2p of catalysts are depicted in Figure 3a, which could be separated into Cu 2p_1/2_ and Cu 2p_3/2_, respectively [24]. The two peaks of Cu 2p_3/2_ located at 933.4 and 934.8 eV corresponded to Cu^+^ and Cu^2+^ species, respectively [1,25]. In addition, the shake-up satellite peak at 942.3 eV was assigned to CuO. According to previous studies [9,26,27], the Cu^+^ species was the main adsorption and activation sites of CO, playing a crucial role in the CO oxidation process. It could be noted that the proportion of the CuFeO_x_ catalyst was 10.2%, which was much lower than that of 17.5% on the CuFeO_x_-100 catalyst. This confirmed that the H_2_ activation could promote the generation of Cu^+^ species, thus increasing the CO conversion.

The O 1s spectra are depicted in Figure 3b. There were two fitted peaks at 529.5 and 531.3 eV, which were attributed to the surface lattice oxygen species (O^2−^) and chemisorbed oxygen species (O_2-_), marked as O_β_ and O_α_, respectively [28]. Generally, the O_α_ species was formed on oxygen vacancy with higher mobility than O_β_ species [4,29,30]. In addition, it was associated with charge unbalance, which was beneficial to the improvement of redox cycle, and an increase in low-temperature activity. It was found that the concentration of O_α_ species increased from 30.1% for the CuFeO_x_ catalyst to 39.6% for the CuFeO_x_-100 catalyst, which implied that the H_2_ activation could accelerate the generation of oxygen vacancy. In addition, it was observed that the peak O_β_ and O_α_ on the CuFeO_x_-100 catalyst shifted towards lower binding energy compared with the CuFeO_x_ catalyst. This may be due to the H_2_ activation weakening the electron-withdrawing ability of Cu and/or Fe species and increasing the surrounding electron cloud density, which resulted in the decrease in binding energy.

For Fe 2p in Figure 3c, two main peaks at 710.8 and 724.0 eV for Fe 2p_3/2_ and Fe 2p_1/2_ were found on the CuFeO_x_ catalyst [31,32], and a satellite peak at 719.4 eV for Fe^3+^ species was observed [33,34]. As for the CuFeO_x_-100 catalyst, peaks at 709.3 and 716.8 eV associated with Fe^2+^ species appeared, which confirmed that the H_2_ activation promoted the Fe^3+^ species reduced to Fe^2+^ species, in agreement with the XRD results. Previous studies had illustrated that the O_2_ could be dissociated to atomic O without a barrier by Fe^2+^ species [35,36]. The activity of CO oxidation was well correlated with the existence and concentration of Fe^2+^ species on the catalyst. Additionally, the coexistence of Fe^2+^ and Fe^3+^ species could establish the redox equilibrium of Cu^+^ + Fe^3+^↔Cu^2+^ + Fe^2+^ on the catalyst surface, which could facilitate the electron transfer between active components to improve the redox cycle. In summary, the H_2_ activation generated Fe^2+^ species, which effectively increased the activity of the CuFeO_x_ catalyst.

### 2.4. Redox Properties

**Figure 4:**
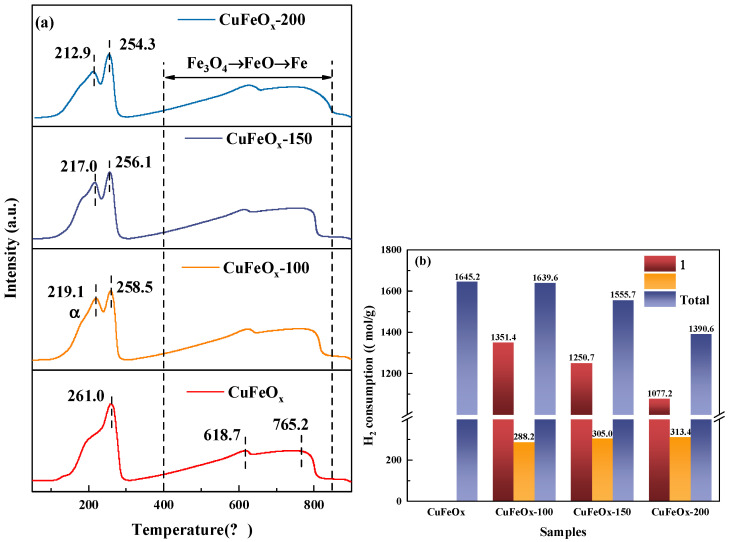
H_2_-TPR profiles (**a**) and the amount of H_2_ consumption (**b**) of catalysts.

The H_2_-TPR was performed to measure the reducibility of catalysts and is shown in Figure 4a. As for the CuFeO_x_ catalyst, the strong peak at 261.0 °C could be assigned to the coreduction of CuO to metallic Cu and Fe_2_O_3_ to Fe_3_O_4_, while the broad peaks in the range of 400 to 800 °C belonged to the sequential reduction of Fe_3_O_4_ to FeO and then to Fe. Compared to the pure Fe_2_O_3_ catalyst [12], the reduction temperature of Fe_2_O_3_ to Fe_3_O_4_ decreased to below 300 °C, indicating that the synergistic effect between Cu and Fe species significantly improved the redox capacity. That is, the Cu species as the active sites facilitated the overflow of H_2_ to Fe_2_O_3_, thus accelerating the reduction to Fe_3_O_4_ at low temperature. Additionally, a shoulder peak appeared on the H_2_-activated catalyst at lower temperature, which may be ascribed to the reduction of CuO or/and Cu_2_O to metallic Cu. This further confirmed that the H_2_ activation promoted the generation of more Cu_2_O. Moreover, the initial reduction temperature on the CuFeO_x_ catalyst was 120 °C, while it shifted to 98 °C on the CuFeO_x_-100 catalyst. The improvement in initial redox property could effectively increase the catalytic activity at low temperature. The Fe species reduced at 400–800 °C had almost no catalytic activity, and activity tests were performed at low temperatures, so it was not involved in the reaction processes. The H_2_ consumption corresponding to the reduction peak below 400 °C was determined based on its integrated area, as depicted in Figure 4b. The total H_2_ consumption on the CuFeO_x_ catalyst slightly decreased from 1645.2 μmol/g to 1639.6 and 1555.7 μmol/g on the CuFeO_x_-100 and CuFeO_x_-150 catalysts, respectively, while it significantly decreased to 1390.6 μmol/g on the CuFeO_x_-200 catalyst. The H_2_ activation at high temperature resulted in part of the active metal oxides being reduced to a metallic state, which may be the main reason for the decline in CO conversion of the CuFeO_x_-200 catalyst.

### 2.5. Temperature-Programmed Studies

**Figure 5:**
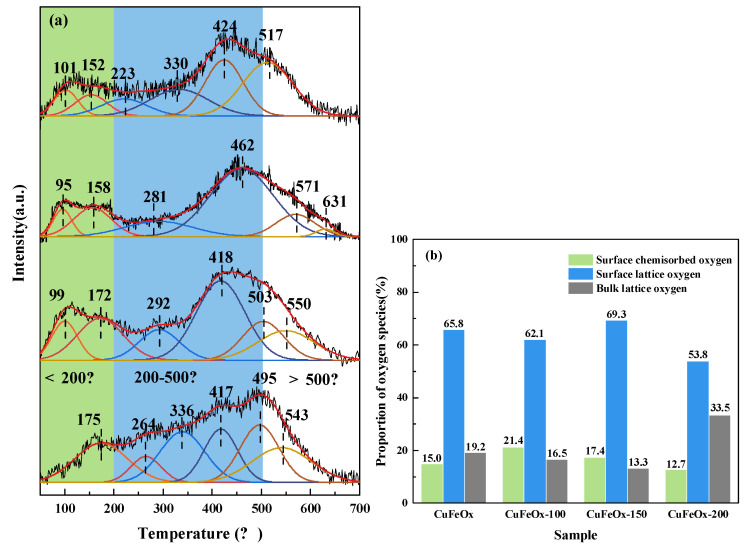
(**a**) O_2_−TPD profiles and (**b**) proportion of oxygen species of catalysts.

The effect of H_2_ activation on the desorption of oxygen species was studied by O_2_-TPD, as exhibited in Figure 5. In Figure 5a, the desorption curves are categorized into three temperature regions [2,37]: (1) The surface chemisorbed oxygen (<200 °C), which is associated with the oxygen vacancy; (2) surface lattice oxygen (200–500 °C); and (3) bulk lattice oxygen (>500 °C). It can be noted that the desorption of surface chemisorbed oxygen on the H_2_-activated catalysts shifted to a lower-temperature position, which implies that its transfer ability was enhanced, and there was favorability for low-temperature CO oxidation. Additionally, the concentration of oxygen species was calculated by the peak area, as displayed in Figure 5b. Compared with the CuFeO_x_ catalyst (15.0%), the proportion of surface chemisorbed oxygen species on the CuFeO_x_-100 (21.4%) and CuFeO_x_-150 (17.4%) catalysts obviously increased, which confirms that the H_2_ activation at a suitable temperature could produce more defects on the catalyst. In addition, the bulk lattice oxygen decreased from 19.2% on the CuFeO_x_ catalyst to 16.5% and 13.3% on the CuFeO_x_-100 and CuFeO_x_-150 catalysts, respectively. This implied an enhancement in the migration of oxygen species from deeper layers to the surface, thereby improving catalytic performance effectively. It can be noted that the percentage of surface chemisorbed oxygen species declined on the CuFeO_x_-200 catalyst (12.7%) compared with the CuFeO_x_ catalyst, which may be due to the overreduction of oxides to the metallic state, which resulted in the decrease in oxygen vacancy.

### 2.6. Reaction Intermediates Analysis

#### 2.6.1. In Situ DRIFT Spectra of CO Adsorption, Ar and O_2_ Purging

**Figure 6:**
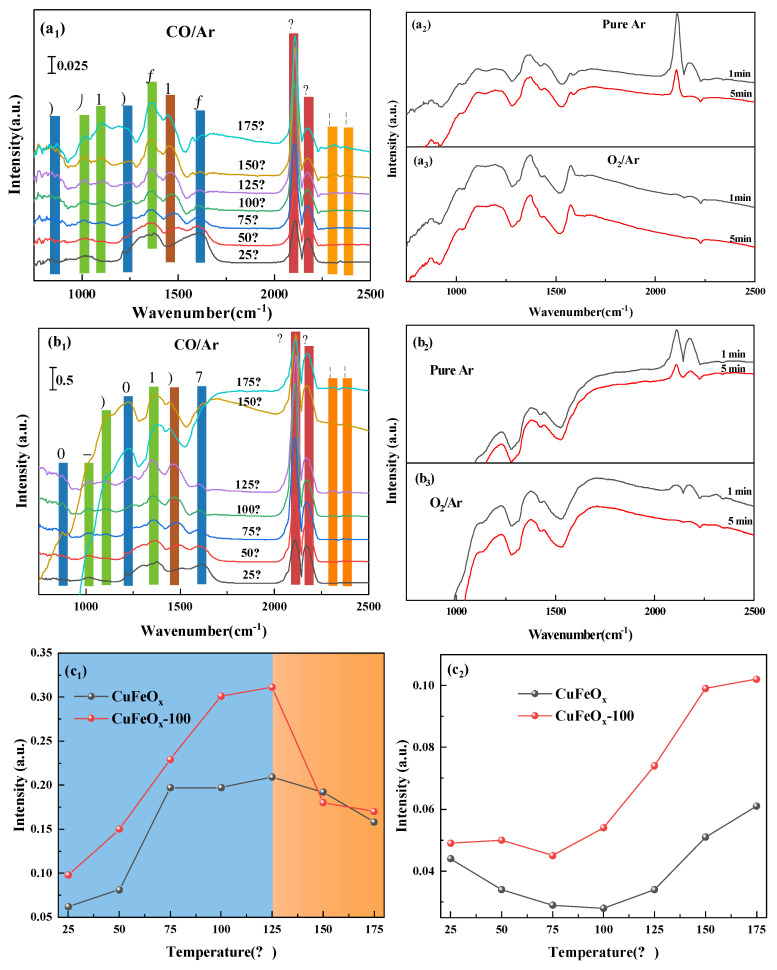
In situ DRFITS spectra of CO adsorption, pure Ar purging, and pure O_2_ purging: CuFeO_x_ (**a_1_**–**a_3_**) and CuFeO_x_−100 (**b_1_**–**b_3_**) catalysts. Behavior of Cu^+^−CO species at 2110 cm^−1^ (**c_1_**) and carbonate species at 1361 cm^−1^ (**c_2_**).( ♣ bicarbonate species, ♦ carbonate species, ♥ monodentate carbonates, ▲ Cu^+^-CO species, ■ CO_2_).

To investigate the active intermediates involved in the CO oxidation reaction, in situ DRIFT was performed. Firstly, the samples were purged in 1% CO/Ar from 25 to 175 °C with temperature rate of 5 min/°C, then switched with pure Ar for 5 min, and finally injected pure 15% O_2_/Ar with 5 min. As seen in Figure 6(a_1_) for the CuFeO_x_ catalyst, the peaks at 2110 and 2172 cm^−1^ associated with Cu^+^-CO species could be found [38,39], and the intensity of Cu^+^-carbonyl species (Cu^+^-CO) strengthened with the increase in temperature initially and then decreased (Figure 6(c_1_)), which indicates that the Cu^+^ species was the main active site for CO adsorption and activation. Additionally, the peaks assigned to carbonate species (1014, 1103, and 1361 cm^−1^) [40], bicarbonate species (838 and 1245 cm^−1^), [41] and monodentate carbonates (1441 cm^−1^) [42] also declined with increasing temperature and were then enhanced, while the peak at 1605 cm^−1^, ascribed to bicarbonate species, decreased [43]. This illustrates that the carbonate species were the active intermediates in the CO oxidation process. In addition, the weak peaks at 2320 and 2356 cm^−1^ belonging to CO_2_ were found even at 25 °C [40], which proves that the CO oxidation can be conducted at ambient temperature, consistent with the results of activity testing. The catalysts were pretreated with pure Ar for 60 min at 300 °C, and the surface adsorbed oxygen was removed completely. Therefore, the CO_2_ originated from the reaction between the Cu^+^-CO species and surface lattice oxygen, and the process followed the MvK mechanism. As described in Figure 6(a_2_), the intensity of Cu^+^-CO species declined after purging with Ar at 175 °C. When O_2_ was introduced (Figure 6(a_3_)), the intensity of the Cu^+^-CO species declined and the peak at 1605 cm^−1^ (bicarbonate species) was enhanced, which illustrates that the consumed active oxygen species can be replenished with gaseous phase O_2_. As for the CuFeO_x_-100 catalyst in Figure 6(b_1_), the type of species was similar to the CuFeO_x_ catalyst, while the intensity of the Cu^+^-CO and carbonate species was stronger (Figure 6(c_1_,c_2_)). This demonstrates that the quantities of Cu^+^ and active oxygen species on the CuFeO_x_-100 catalyst were higher than those of the CuFeO_x_ catalyst, in agreement with the XPS and O_2_-TPD analysis. Furthermore, the intensity of Cu^+^-CO species began to reduce after 125 °C both for CuFeO_x_ and CuFeO_x_-100 catalyst (Figure 6(c_1_,c_2_)), which suggests that the reaction had entered a phase of rapid progress. It was found that the reduction range of intensity for Cu^+^-CO species on the CuFeO_x_-100 catalyst was larger than that on the CuFeO_x_ catalyst after 125 °C (Figure 6(c_1_)), which indicates that the Cu^+^-CO species was more active.

#### 2.6.2. CO+O_2_ Coadsorption

**Figure 7:**
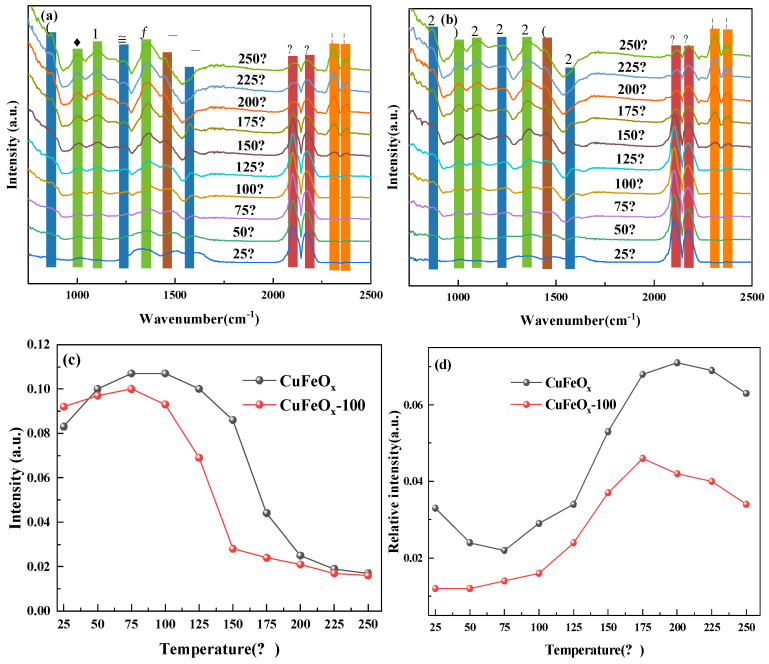
In situ DRFITS spectra of CO+O_2_ coadsorption on CuFeO_x_ (**a**) and CuFeO_x_−100 (**b**) catalyst. Behavior of Cu^+^−CO species at 2110 cm^−1^ (**c**) and carbonate species at 1361 cm^−1^ (**d**). ( ♣ bicarbonate species, ♦ carbonate species, ♥ monodentate carbonates, ▲ Cu^+^-CO species, ■ CO_2_).

The in situ DRFITS of CO+O_2_ coadsorption is displayed in Figure 7. As depicted in Figure 7a,b, the species and variation trend of intensity with temperature was similar in CO atmosphere both for CuFeO_x_ and CuFeO_x_-100 catalysts. As illustrated in Figure 7c, the intensity of the Cu^+^-CO species on the CuFeO_x_ and CuFeO_x_-100 catalysts was decreased to 100 and 75 °C compared with CO atmosphere, respectively, which proved that the O_2_-riched atmosphere was conducive to the CO oxidation. In addition, the Cu^+^-CO species on the CuFeO_x_-100 catalyst was stronger than the CuFeO_x_ catalyst at 25 °C and lower at higher temperature, which further confirms that the Cu^+^-CO species with H_2_ activation was more active. As displayed in Figure 7d, the carbonate species both on CuFeO_x_ and CuFeO_x_-100 catalysts was lower than in CO atmosphere, which implies that the presence of O_2_ can promote the decomposition of carbonate species. In contrast to the CO atmosphere (Figure 6(c_2_)), the strength of carbonate species on the CuFeO_x_-100 catalyst was higher than that on the CuFeO_x_ catalyst (Figure 7d), indicating that carbonate species were more easily decomposed on the CuFeO_x_-100 catalyst in reaction to atmosphere. Interestingly, it was found that the intensity of the carbonate species started to decrease and was accompanied by the generation of CO_2_ sharply after 175 °C, which suggests that the CO oxidation process was conducted through the L-H mechanism.

## 3. Discussion

Based on the above results, the H_2_ activation with appropriate temperature can efficiently increase the activity of CuFeO_x_ catalyst for CO oxidation (Figure 1a). XRD analysis (Figure 2) showed that the pure Fe_2_O_3_ had good crystallization and large grain size, and the addition of Cu species can decrease the crystallization. In addition, some peaks attributed to CuO disappeared with the H_2_ pretreatment at 100 and 150 °C, confirming that the CuO was reduced to Cu_2_O. After pretreatment at 200 °C, the metal Cu was produced. The specific surface area increased slightly with the introduction of Cu species (Table 1), which could promote the dispersion of active components and adsorption of reaction gas. Following H_2_ activation, there was minimal change observed in the specific surface area, average pore diameter, and pore volumes of the catalysts. This suggests that the surface physical structure may not be the primary factor contributing to the improvement in catalytic performance. Furthermore, the XPS results (Figure 4) illustrate that the percentage of Cu^+^ species increased on the H_2_-activated catalyst, which could offer more active sites for CO, and more O_α_ species were produced with the H_2_ pretreatment, which proved that more oxygen vacancy was formed. This could facilitate the activation of oxygen species and the circulation of the reaction. In addition, the Fe^2+^ species was generated, in agreement with the XRD results, which was beneficial to the dissociation of O_2_ to oxygen atoms. Moreover, the H_2_-TPR analysis indicated that the moderate H_2_ activation could enhance the reducibility of the CuFeO_x_ catalyst, which could increase the catalytic activity for CO oxidation. However, the H_2_ activation at high temperature led to the decrease in H_2_ consumption and redox property. The O_2_-TPD results indicate that H_2_ activation promoted the migration of oxygen species from deeper layers to the surface. As a result, the concentration of both surface chemisorbed oxygen and surface lattice oxygen increased, potentially providing more active oxygen species for CO oxidation.

In situ DRIFTS experiments were performed to investigate the reaction mechanism. The results indicate that the predominant reaction pathway followed the MvK mechanism at low temperatures (<175 °C). The specific procedure was as follows: (1) Firstly, the CO combined with Cu^+^ to form Cu^+^-CO species (Equation (1)). The H_2_ activation increased the quantity of Cu^+^ species, which enhanced the reaction procedure of Equation (1). Subsequently, the Cu^+^-CO species reacted with surface lattice oxygen to directly generate CO_2_ and leave the oxygen vacancies, as displayed in Equation (2). Finally, the oxygen vacancies were resupplemented by gas O_2_, and the cyclic process was completed (Equation (3)).

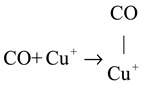
(1)

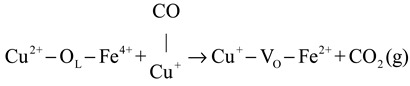
(2)

(3) $${\mathrm{Cu}}^{+}-V_{O}-Fe^{2+}+1/2O_{2}\to {\mathrm{Cu}}^{2+}-O_{L}-Fe^{3+}$$

**Scheme 1:**
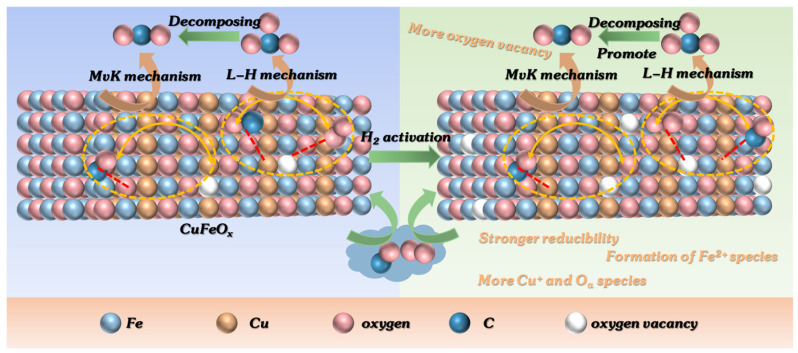
Mechanism model of CO oxidation on CuFeO_x_ and CuFeO_x_-100 catalysts.

CO oxidation on the CuFeO_x_ catalyst at high temperatures (>175 °C) proceeded via both the MvK and the L-H mechanism, with the latter described in Equations (4)–(6). The gas O_2_ was captured by oxygen vacancy and formed chemisorbed oxygen species, as shown in Equation (4). The H_2_ soft pretreatment increased the concentration of oxygen vacancy on the CuFeO_x_ catalyst, which promoted the process of Equation (4). Then, the chemisorbed oxygen species reacted with Cu^+^-CO species (Equation (1)) to generate carbonate species, as exhibited in Equation (5). The carbonate species decomposed into CO_2_, as described in Equation (6). The H_2_ activation accelerated the decomposition of carbonate species, which alleviated the accumulation of carbonate occupying the active sites and improved the efficiency of CO oxidation. Based on the above, a possible reaction model is proposed and is illustrated in Scheme 1.

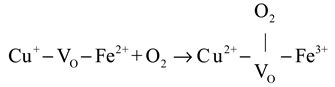
(4)

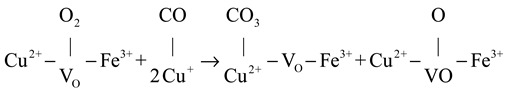
(5)

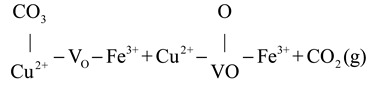
(6)

## 4. Materials and Methods

### 4.1. Preparation of Catalyst

The catalysts were synthesized using the coimpregnation method. Cu(NO_3_)_2_·3H_2_O and Fe(NO_3_)_3_·9H_2_O were dissolved in ionized water with the molar rate of Cu:Fe = 1:2. Then, the solution was stirred by the magnetic force and heating in a water bath until the water was completely evaporated. Subsequently, the solid was dried for 24 h at 80 °C. Finally, it was calcined at 450 °C for 4 h. The obtained sample was donated as a CuFeO_x_ catalyst. In the H_2_ activation stage, the tube furnace was vacuumed, and then CuFeO_x_ was heated to 100 °C under a N_2_ protective atmosphere. Finally, 10% H_2_/He was pretreated for 0.5 h. The obtained sample was marked as the CuFeO_x_-100 catalyst, and the samples pretreated with 10% H_2_/He at 150 and 200 °C were marked as the CuFeO_x_-150 and CuFeO_x_-200 catalyst, respectively.

### 4.2. Catalytic Performance Test

The catalytic activity was evaluated on a fixed-bed quartz reactor with an inner diameter of 8 mm, and 300 mg of catalyst was used for each test. A type K thermocouple was inserted into the catalyst bed to monitor the reaction temperature. The composition of the simulated gas mixture was 1% CO, 10% O_2_, and N_2_ balance. The total gas flow rate was maintained at 300 mL/min, while the reaction temperature was incrementally increased from 25 to 300 °C at a heating rate of 5 °C/min. Test points were recorded at 25 °C intervals, and the CO concentration at each point remained constant for 30 min. Gas concentrations at the inlet and outlet were measured using an online gas chromatograph. The CO conversion rate was determined by employing Equation (7):

(7) $$\mathrm{CO}\;conversion(\%)=\frac{{\left[CO\right]}_{in}-{\left[CO\right]}_{out}}{{\left[CO\right]}_{in}}\times 100\%$$

where the [CO]_in_ and [CO]_out_ represent the concentration of CO at the inlet and outlet, respectively.

### 4.3. Catalyst Characterization

The physicochemical properties of catalysts were charactered with XRD, BET, XPS, H_2_-TPR, O_2_-TPD, and in situ DRIFTS, and can be found in the supporting information (SI).

## 5. Conclusions

In this work, the effect of H_2_ activation on the performance of the CuFeO_x_ catalyst for low-temperature CO oxidation was investigated. It was found that the soft H_2_ pretreatment could effectively improve the activity of the CuFeO_x_ catalyst, and the catalyst activated at 100 °C displayed the highest performance, which corresponded to 99.6% CO conversion at 175 °C. The influence of H_2_ activation on the surface physical structure was negligible. The H_2_ activation enhanced the reducibility of CuFeO_x_ catalyst, thus improving the low-temperature activity. In addition, the H_2_ activation generated the Fe^2+^ species, which was beneficial to the dissociation of O_2_. Furthermore, the amount of O_α_ species and oxygen vacancy increased after H_2_ activation, which increased the cycle efficiency of the CO oxidation process. Moreover, the migration of oxygen species from the deep layer to the surface was promoted, which could provide more active oxygen species for CO oxidation. The in situ DRFITS demonstrated that the CO oxidation pathway mainly involved the MvK mechanism at low temperature, and both MvK and L-H mechanisms at high temperature. The Cu^+^-CO and carbonate species were the main active intermediates, and the H_2_ activation increased the amount of Cu^+^ species and accelerated the decomposition of carbonate species, which increased the activity of the CuFeO_x_ catalyst.

## Figures and Tables

**Table 1:** Catalysts’ BET specific surface area, pore volume, and average pore size.

Sample	Specific Surface Area (m^2^/g)	Average Pore Diameter (nm)	Total Pore Volume (cm^3^/g)
Fe_2_O_3_	9.85	14.96	0.037
CuFeO_x_	11.06	14.98	0.041
CuFeO_x_-100	12.60	13.36	0.042

**Table 2:** XPS data of the catalysts.

Sample	Cu 2p (%)		Fe 2p (%)		O 1s (%)	
	Cu^+^	Cu^2+^	Fe^2+^	Fe^3+^	O_α_	O_β_
CuFeO_x_	17.5	82.5	/	100	30.1	69.9
CuFeO_x_-100	10.2	89.8	17.9	82.1	39.6	60.4

## Data Availability

Data are contained within the article and Supplementary Materials.

## Supplementary Materials

The following supporting information can be downloaded at: https://www.mdpi.com/article/10.3390/molecules29143347/s1, Figure S1: N2 adsorption–desorption isotherms. Figure S2: The SEM profiles of catalysts.
