# Supplementary material for: Unveiling the Promoting Mechanism of H2 Activation on CuFeOx Catalyst for Low-Temperature CO Oxidation
Source: Molecules. 2024 Jul 17;29(14):3347. doi: 10.3390/molecules29143347 (PMC11279789; doi:10.3390/molecules29143347)
Supplement: Supplementary file 1 [file molecules-29-03347-s001.zip › molecules-3059074-supplementary.pdf]

S1. Experimental details

The Brunauer-Emmett-Teller (BET) surface area, pore volume and pore size distribution of the catalysts were performed with N_2 adsorption/desorption under -196 °C on NOVA 4200e.

The X-Ray diffraction (XRD) of the samples was measured by a PANalytical X'Pert-Pro X-ray diffractometer with a Cu-Kα monochromatized radiation source in the range of 10° to 90°, with a scan rate of 4°/min and a step 0.02°.

XPS spectra are measured on the An ESCALAB 210 (VG Scientific) to obtain the corresponding peak intensity of Fe 2p, Cu 2p and O 1s of catalysts.

The H_2 temperature programmed reduction (H_2-TPR) of the catalysts were determined on MICROMERITICS AUTOCHEM II. The sample was pretreated in pure He at 300 °C for 60min. After cooling to 50 °C, the sample was heated to 900 °C at the rate of 10 °C/min under 5%H_2/He mixtures.

The O_2 temperature-programed desorption (O_2-TPD) was also examined on MICROMERITICS AUTOCHEM II. Each sample was pretreated in pure He at 300 °C for 60 min. The samples were exposed to O_2 (10%, N_2 as the balance) at 50 °C for 1 h, and then pure He was introduced to purging the samples for 0.5 h. TPD curves were recorded with temperature raising at a heating rate of 10 °C/min.

In situ diffuse reflectance infrared Fourier transform spectroscopy (DRIFTS) were conducted using a Nicolet iS50 FTIR spectrometer with a MCT detector at a spectral resolution of 4 cm^{-1} and accumulation of 32 scans. Prior to the experiment, the prepared catalysts could be purged at 300 °C for 1 h in N_2 atmosphere, and background spectra were collected at the target temperature during the cooling process. The reaction conditions were controlled as follows: 100 mL/min total flow rate, 10% O_2, 1% CO and N_2 balance. The spectra collected were able to explore the reaction mechanism at different temperature between the catalysts and reaction gases. The spectra collected were able to explore the reaction mechanism at different temperature between the catalysts and reaction gases.

S2. Supplemental data

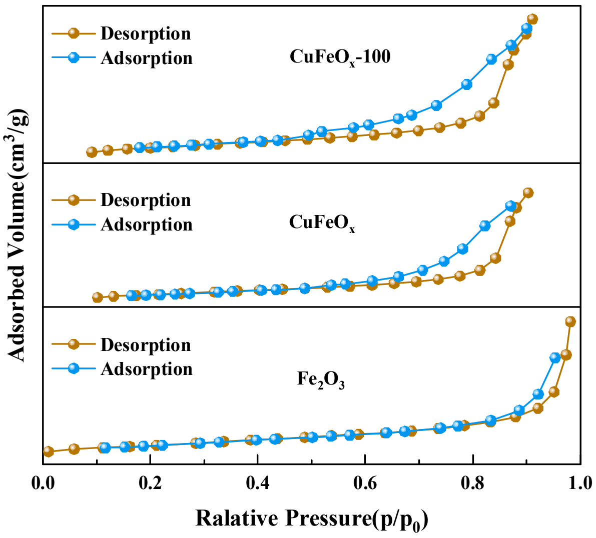

Figure S1 N_2 adsorption–desorption isotherms.

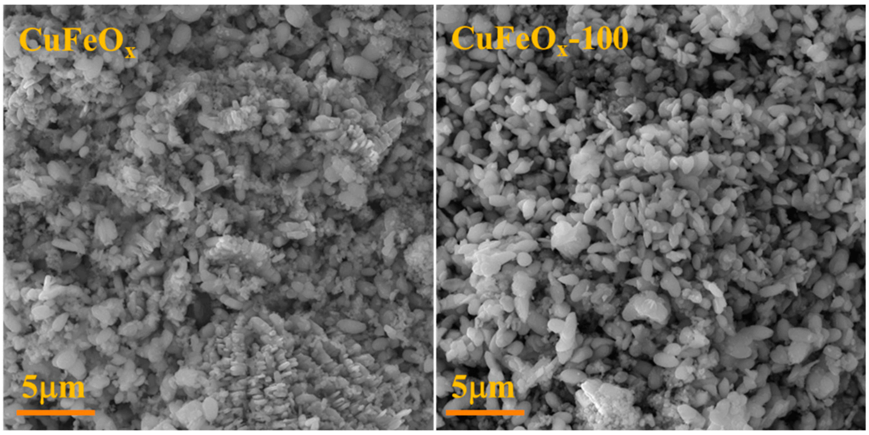

Figure S2 The SEM profiles of catalysts.
